# Usability engineering in practice: developing an intervention for post-stroke therapy during a global pandemic

**DOI:** 10.1080/03091902.2022.2089257

**Published:** 2022-08-24

**Authors:** Avril D. McCarthy, Louise Moody, Mark L. Reeves, T. Jamie Healey, Tim Good, Lise Sproson, Adewale Adebajo, Wendy Tindale, Krishnan Padmakumari Sivaraman Nair

**Affiliations:** a Clinical Engineering, Sheffield Teaching Hospitals NHS Foundation Trust, Sheffield, UK; b NIHR Devices for Dignity MedTech Co-operative, Sheffield Teaching Hospitals NHS Foundation Trust, Sheffield, UK; c Centre for Arts, Memory and Communities, Coventry University, Coventry, UK; d Department of Neurology, Sheffield Teaching Hospitals NHS Foundation Trust, Sheffield, UK; e Department of Rheumatology, Barnsley Hospital NHS Foundation Trust, Barnsley, UK

**Keywords:** Stroke, stroke therapy, usability engineering, SHAPES, intervention

## Abstract

This paper provides an overview of the usability engineering process and relevant standards informing the development of medical devices, together with adaptations to accommodate situations such as global pandemics where use of traditional face-to-face methods is restricted. To highlight some of those adaptations, a case study of a project developing a novel electronic rehabilitation device is referenced, which commenced in November 2020 amidst the COVID-19 pandemic. The Sheffield Adaptive Patterned Electrical Stimulation (SHAPES) project, led by Sheffield Teaching Hospitals NHS Foundation Trust (STH), aimed to design, manufacture and trial an intervention for use to treat upper arm spasticity after stroke. Presented is an outline and discussion of the challenges experienced in developing the SHAPES health technology intended for at-home use by stroke survivors and in implementing usability engineering approaches. Also highlighted, are the benefits that arose, which can offer easier involvement of vulnerable users and add flexibility in the ways that user feedback is sought. Challenges included: restricted travel; access to usual prototyping facilities; social distancing; infection prevention and control; availability of components; and changing work pressures and demands. Whereas benefits include: less travel; less time commitment; and greater scope for participants with restricted mobility to participate in the process. The paper advocates a more flexible approach to usability engineering and outlines the onward path for development and trialling of the SHAPES technology.

## Introduction

Whether healthcare technology is developed for use by healthcare professionals, or by a patient or carer, usability is important to ensure it is safe and easy to use. Usability can be defined as: *“The extent to which a system, product or service can be used by specified users to achieve specified goals with effectiveness, efficiency and satisfaction in a specified context of use.”* [1]

Usable products, systems and interventions increase the likelihood of appropriate and sustained usage, thereby supporting healthy behaviours and outcomes [2]. By identifying and addressing usability and acceptability problems early in the development process, the required resources, costs and time are reduced [3]. Unfortunately there are many examples of health technologies, that are clinically effective at a functional level, but because of design and usability issues are not accepted by users and therefore do not realise the intended health benefits [4–6].

The application of usability engineering is fundamental in developing effective and safe medical devices, systems, services and interventions to optimise patient care, health, and well-being. The UK Medicines and Healthcare products Regulatory Agency (MHRA) define “usability engineering” as the process of achieving usable products that address user needs and fit with their practices [7]. This is especially important where the intended user will be the recipient of therapy, or care management delivered by the technology. The importance of usability engineering is increasingly recognised and emphasised by regulators in their promotion of best industry practices, safety and quality in device development.

Table 1 outlines some of the UK, European and International standards and recommendations relating to medical devices, risk management, electrical safety and human factors engineering – areas that are commonly intertwined in ensuring safe and acceptable device usability. Within Table 1 the ISO 14971 standard [8] relates to risk management and this should be applied during the life cycle of all forms of medical device. Depending on whether the device is electrically powered and/or employs software, will also influence the selection and use of other specific standards highlighted in Table 1 such as the IEC 60601 family for medical electrical equipment or IEC 62304 [11] for medical device software. IEC 62366 [10] relates specifically to medical device usability during normal usage. However, IEC 60601-1-6 [12], which also focuses on usability, may be more appropriate to employ when developing electrical medical devices. The context of usage is also important, and within the 60601 family is 60601-1-11 [13], which focuses on intended medical device use in the home environment. Again referenced in Table 1, the UK’s medical device regulator (the MHRA), the European Commission and the American National Standards Institute (ANSI) have provided guidance, recommendations and updated medical device regulations centring on usability engineering and human factors. Thus reflecting the importance of achieving effective usability to reduce user risk and deliver safe devices able to meet regulatory compliance requirements.

**Table 1.** Relevant standards, regulations and recommendations related to usability engineering of medical devices.

Organisation	Role	Example recommendations
Medicines and Healthcare products Regulatory Agency (MHRA)	Responsible for ensuring the safety, quality and effectiveness of medicines, medical devices and blood components for transfusion in the UK	Recommendations on Human Factors and usability engineering during medical device development. Recommend submission of a Human Factors summary report and post-market surveillance for human factors and ergonomic issues [7].
International Organisation for Standardisation (ISO)	Develop and publish international standards.	ISO 14971 [8] relates to the application of risk management of medical devices and is the overarching standard to employ in developing safe and effective devices and, as such, infers an expectation to assess safe usability through the application of related usability standards. The current 2019 3rd edition, increases emphasis on defining the benefit and state- of-the-art of the device as it relates to residual risk, introduced a new definition of “*reasonably foreseeable misuse”* – (emphasizing the *context* of such use in assessing whether it can be used safely)- and increased focus on gathering post-market user feedback
International Electro-technical Commission (IEC)	Prepares and publishes international standards for all electrical, electronic and related technologies	IEC (or BS EN) 62366 specifies a process by which a manufacturer can analyse, specify, develop and evaluate the usability of a medical device to ensure safety and mitigate risks associated with correct use, and use errors, within the scope of normal use [9,10]. IEC (or BS EN) 62304 defines the “life cycle requirements” for medical device software and describes a set of processes, activities, and tasks to establish common framework for medical device software life cycle processes, when software is itself a medical device, or when software is an embedded or integral part of the final medical device [11]. IEC (or BS EN) 60601 refers a family of standards relating to the medical electrical equipment. Part 1 (60601-1) relates to the **basic safety and essential performance** for all medical electrical equipment. Part of the same family, IEC (or BS EN) 60601-1-6 is an updated collateral standard specifically intended to support the **usability** of medical electrical equipment. [12] Considering the context of usage, the collateral standard IEC 60601-1-11:2015 (or BS EN BS EN 60601-1-11:2015 + A1: 2021 for those in the UK/EU) considers the basic safety of medical electrical equipment when used in the **home environment** [13].
European Commission	The European regulatory framework ensures the safety and efficacy of medical devices and facilitates patient access to devices in the European market.	The EU Medical Device Regulation (2017/745) [14] increased focus on usability compared to the former EU Medical Device Directive (93/42/EEC) in recognition of safety issues arising related to usability and an increasing shift to more lay-user managed medical devices [15]. There is an emphasis on specifying the users of a device, and ensuring that the human factors issues that influence use and interactions are fully understood. It aims to ensure the medical device is designed with the end-users and that their cognitive and physical capabilities and limitations are understood and taken into account through the design. *N.B. MHRA consultation on the future requirements for UKCA marking following the UK’s exit from the EU is underway.*
American National Standards Institute (ANSI)	Promote and facilitate voluntary consensus standards and conformity assessment systems to enhance US global business competitiveness and US quality of life.	ANSI/AAMI HE 75:2009, [16]. Human factors engineering – design of medical devices. Provides detailed guidance on how to perform specific human factors analyses and provides a wealth of design principles

As well as the focus on the usability of medical devices, there has also been a response to the wider use of technologies by patients and lay-caregivers at home, who with limited skills or training, need to operate often complex devices. While home usage should be considered as one context of usability, often such important use contexts are overlooked in the medical device field. A White Paper from the British Standards Institution (BSI) [17] gives guidance on engaging stakeholders in the home market, reinforcing that usability is particularly important for safety and effectiveness for home usage, and particularly when introducing a complex or unfamiliar technology. This is further supported by the IEC 60601-1-11 standard [13] referenced in Table 1.

### End-user involvement

Central to usability engineering is designing, and developing with the end-users, while understanding and taking into account their capabilities and limitations through the design and engineering development. Usability can be considered using different approaches [18–20], for example through:

1. Co-design: developing with and for stakeholders;
2. User feedback gained from usage experience from deployment of a similar device
3. User feedback during the design process: gaining iterative feedback on designs as they evolve, as well as a final prototype or device;
4. Expert assessment: drawing on networks of healthcare experts, scientists and academics to assess the solution against the clinical context;
5. Usability testing with end users: assessing ease of use and acceptability.

**Figure 1.**
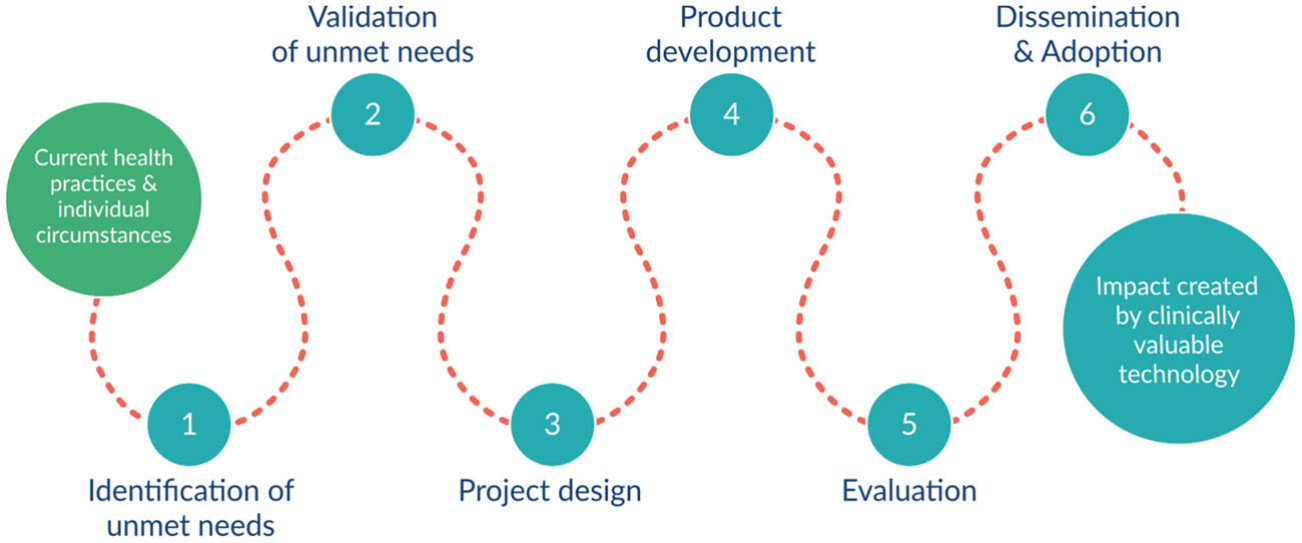
NIHR Devices for Dignity MIC innovation process [22].

User-centred and co-design approaches should seek to support regulatory compliance whilst also encouraging design creativity and innovation. The clinical engineering department at Sheffield Teaching Hospitals NHS Foundation Trust (STH) works within a certificated ISO 13485:2016 quality management system [21]. As part of this quality process for developing clinical investigational and medical devices, user needs are considered and regulatory requirements met throughout device development to ensure that the technology achieves the intended health and clinical value. The high-level innovation process developed and implemented by the NIHR Devices for Dignity MedTech Co-operative (D4D MIC), – illustrated in Figure 1 [22], is based loosely on ISO 13485 and positions the user at the heart of the iterative technology development process.

### Challenges of usability engineering

There are many recognised challenges to involving users in the development of technology. Typical examples include: time and resources, ensuring access to representative users, managing user expectations (especially where there are fundamental engineering or regulatory constraints), overcoming the technological language barrier, balancing competing and often conflicting requirements, dealing with sensitive and ethical challenges, and supporting a range of complex needs [20,23–25].

During the COVID-19 pandemic, usability engineering within a health context has been particularly challenging. The pandemic led to competing demands on NHS staff including:

- Increased immediate healthcare delivery, in parallel with,
- Urgent research and development to advance COVID-19 diagnosis, treatment and management [26–28].

This led to clinical science and engineering capacity becoming strained within healthcare environments, especially for those within the UK’s National Health Service (NHS).

In the remainder of this paper we will outline some of the specific challenges experienced by the authors in developing a new medical technology during the pandemic as part of the SHAPES project. Public patient involvement (PPI) and engagement methods were employed as part of the usability engineering process. A wider review of PPI methodologies is provided in a separate paper within this special journal edition and so is not discussed in depth in this paper.

## SHAPES: a case study example

The SHAPES project: “*A new therapy for post-stroke arm spasticity: Sheffield Adaptive Patterned Electrical Stimulation (SHAPES) - a co-designed system improvement followed by a powered multi-arm randomised control trial”,* was funded by the National Institute for Health Research (NIHR) Invention for Innovation (i4i) programme (NIHR201642: 2020-2024).

### Stroke and upper limb spasticity: the unmet need

Approximately 85,000 people per year in the UK experience a stroke, about 60% survive to one year with a disability [29,30]. The number of survivors is expected to rise by 123% in the next 20 years [30]. A key priority of the NHS England Long Term Plan is to increase the availability and quality of post-stroke rehabilitation, so that it is responsive to patient demand and supports good recovery at home [31].

Research suggests 17–43% of stroke survivors experience long-term muscle over-activity resulting in excessive stiffness termed “*spasticity*” [30–32]. Spasticity restricts joint range and mobility, limiting free and controlled movement of a limb or limbs. Of the upper limb joints, the elbow is affected most frequently, though other joints such as shoulder, wrist and fingers can also be impacted negatively. This can become painful making it hard to perform common activities of daily living and often results in reduced independence and carer assistance [33]. On average the NHS and social care costs are £22,175 per survivor at year 1 [29], with spasticity quadrupling direct costs [34].

Conventional therapy is typically physiotherapy plus oral medications and potentially injectable drug therapies. However, outcomes are unsatisfactory for about 35% of stroke survivors, and not cost-effective. The lack of appropriate or timely treatment can cause limb pain, muscle atrophy, joint contracture and deformation, which can adversely affect recovery of normal activities after stroke [35,36].

### The evolution of SHAPES

**Figure 2.**
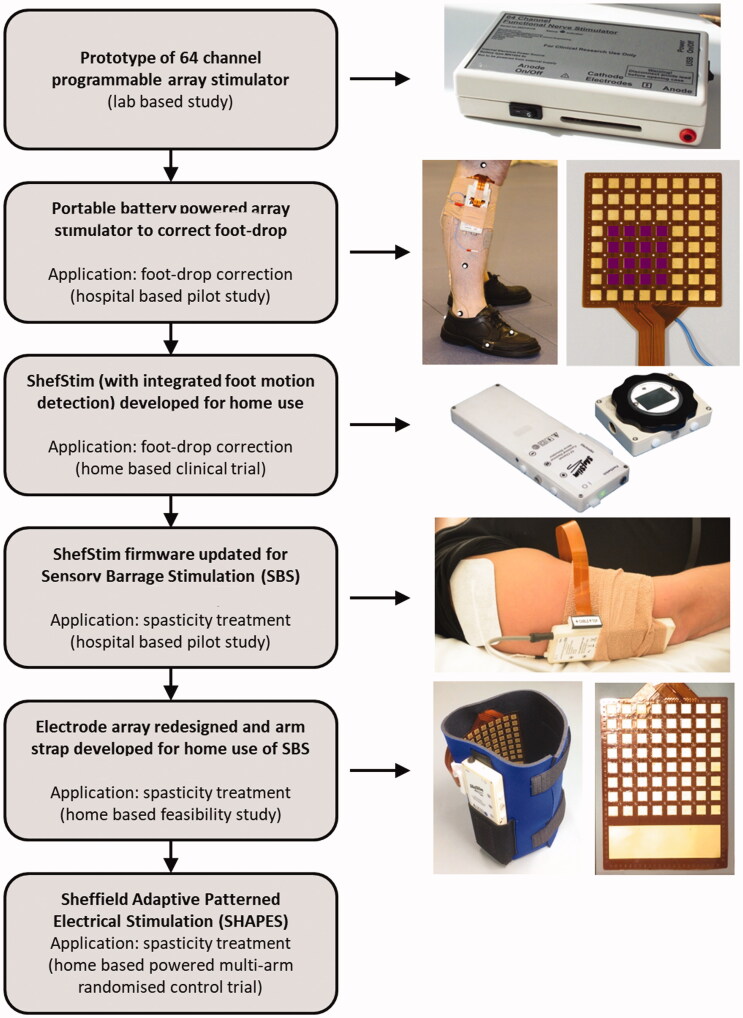
Development pathway of SHAPES.

Previous research by STH had developed and explored the use of a complex form of transcutaneous electrical nerve stimulation (TENS), termed “Sensory Barrage Stimulation” (SBS), for treating post-stroke upper limb spasticity. If effective, SBS would allow more therapy time at home, avoid anti-spasticity medications, with reduced health and social care utilisation through improved capacity and independent living. SBS evolved into the SHAPES form of electrical stimulation used within this case study (see the evolution of SHAPES outlined in Figure 2).

Although similar to TENS, the stimulation levels delivered by *functional* electrical stimulation (FES) systems are intended to induce targeted muscle contraction in restoring movement functions. Typical commercial FES systems provide a single channel of stimulation. The Sheffield team at STH had developed a novel 64-channel electrical stimulator, initially intended to aid patients in optimising their FES therapy for correcting foot-drop – common after stroke or in those with multiple sclerosis [37]. The FES foot-drop device was re-developed into a 64-channel electrical stimulator to deliver sensory stimulation (SBS) for treatment of upper limb spasticity. It delivers a varying (spatially and temporally) pattern of stimulation at sub-motor threshold level over a multitude of electrodes/adjacent locations rather than *via* fixed electrodes [38].

The new SHAPES system consists of a small box containing the stimulator electronics (ShefStim APS), linked to an 8 by 8 array of 64 electrodes, overlaid with a bespoke hydrogel layer, and worn on the upper arm with a flexible “sleeve”. It sends tiny electrical pulses *via* the electrodes and, by programming the system to apply this stimulation to different groups of electrodes at different times; it produces moving patterns of sensation – similar to being stroked. Other devices like TENS (Transcutaneous Electrical Nerve Stimulation) work similarly, but only produce a sensation under the electrodes at 2 static locations resulting in sensations akin to repeated “tapping” over the same spot. We hypothesise the therapeutic effect of SHAPES will be greater because the stimulation is delivered over a larger area, and varies in nature, thus preventing habituation of the nervous system to repetitive identical stimulation.

An in-hospital pilot trial showed both TENS and SHAPES to have a therapeutic effect, but only SHAPES had a continued benefit one-hour post-treatment [38]. A further feasibility study explored community-based caregiver delivery of treatment to patients with post-stroke upper limb spasticity. Results showed that participants could successfully deliver 80% of the planned treatment sessions, and without experiencing significant adverse effects [39]. Participants within this feasibility study made suggestions to improve ease-of-use of the system by the user (and/or carer) by facilitating single-handed application of the wearable sleeve for independent use, and to offer a remote control to supplement the on-board stimulator controls when worn on the arm [39].

In 2020, STH was awarded NIHR i4i funding to further advance the design and manufacture of a self, or carer-managed intervention, with a Randomised Control Trial (RCT) planned to start early in 2022. The project commenced in November 2020 during the COVID-19 pandemic and second national lockdown in the UK. The collaborative team consists of NHS based researchers, academic researchers and industry sub-contractors distributed across England.

### Pre-pandemic development and usability

During the studies that preceded the SHAPES project (pre-2020), traditional usability methods were employed. Several face-to-face meetings with differing focus areas were held, attended by members of the Sheffield Stroke PPI group and informed by the clinical teams.

During pre-2020 studies [38–40] that preceded the SHAPES project, traditional usability methods were employed – these are illustrated and summarised in brief for contrast.

Several face-to-face meetings with differing focus areas were held, attended by 10 lay advisory group members (from of Sheffield and Salford Stroke PPI groups) and informed by the clinical teams.

**Figure 3.**
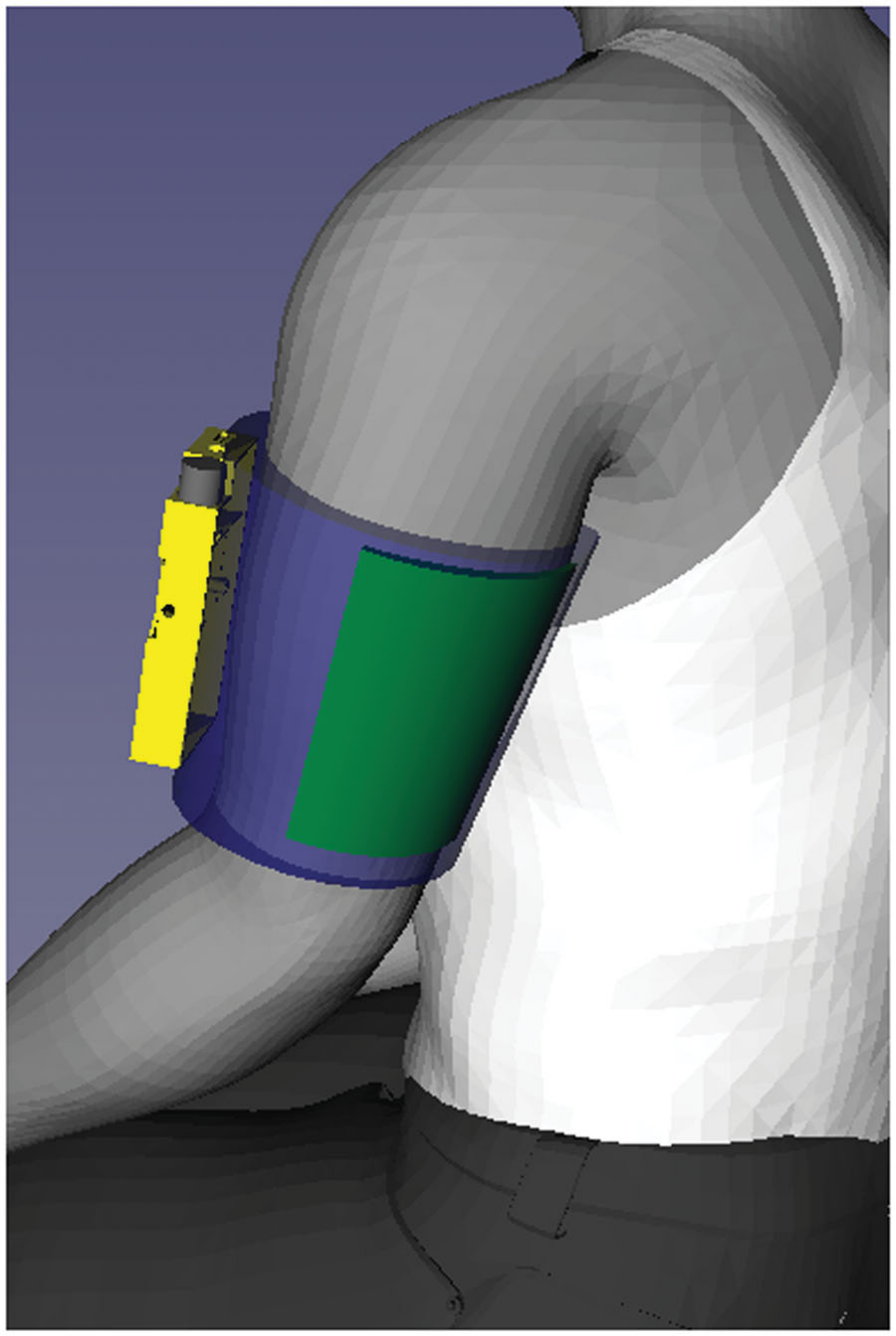
Illustration of the relative size and positioning of SHAPES system in order for the treatment to be administered (stimulator in yellow, electrode array in green, arm sleeve in blue).

**Figure 4.**
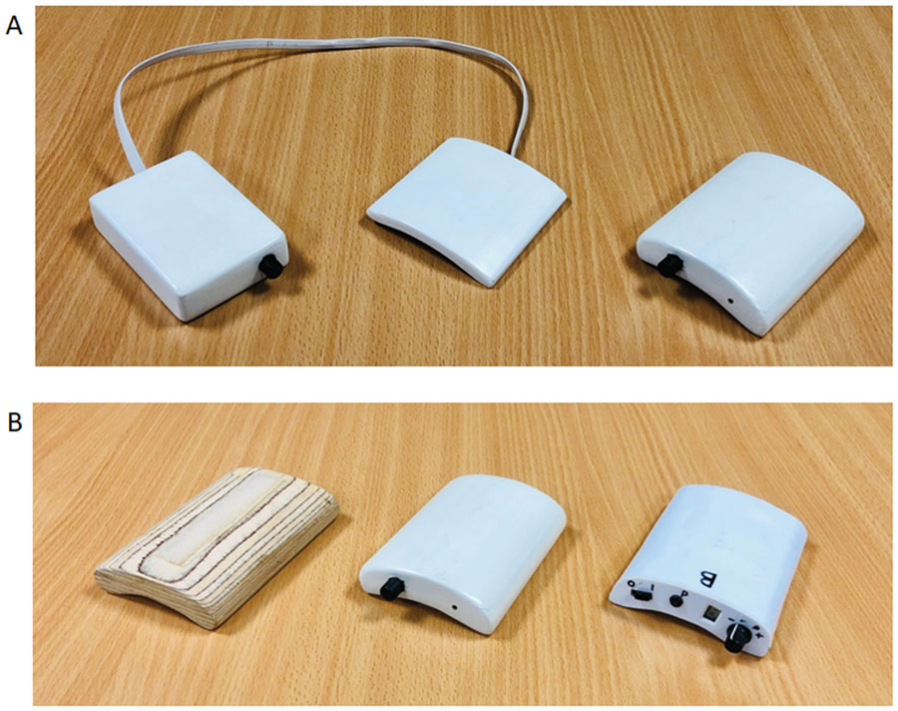
Models created for pre-pandemic usability studies of showing different shapes and forms for the stimulator (A). Models incorporating different control options were later produced (B) [40].

The graphic (Figure 3) provides an overview of how the SBS (now SHAPES) device is worn. To assess the usability of the SBS stimulator and its accessories, a range of design artefacts were developed. To gauge fit, comfort and reach to controls when in use, one-off models (shown in Figure 4) of suitably weighted non-functional stimulators in different shapes and forms had been fashioned from wood and painted wood. The group format allowed the different versions to be handled and passed around for collective discussion focussing on, various issues like positioning of the electrode array within the sleeve and placement and type of control mechanisms (Figure 4).

Informed by the previous studies [38–40] and advice from stroke survivors in planning the NIHR grant application, the SHAPES team deemed it important to include an arm-sleeve re-design element. With many survivors losing the use of one arm and hand after a stroke, facilitating single-handed donning (putting on) and doffing (taking off) was a project design aspiration. Other issues considered were connection of the array to the stimulation box and inclusion of a remote control for ease of using the stimulator. To develop sleeve design options, different types of materials, forms, options to hold the stimulator in place, and tightening mechanisms were discussed.

## Prototyping and usability engineering during the pandemic

The following section outlines some of the COVID-19 pandemic challenges experienced and how these were addressed to accommodate those restrictions – during the period November 2020 - July 2021. Although University ethical approval was in place, advice was sought from the Public Involvement Lead of the Health Research Authority regarding the need for NHS ethical approval before commencing usability activities with stroke survivors who were not part of the project team [41]. As the usability participants were to be asked to provide preference and usability insights only, they were not deemed to be research participants and we were informed that NHS ethical approval was not required.

### Prototype development

Travel restrictions plus restricted access, or, in some circumstances no access, to workplaces as a result of the pandemic, affected development and collaborative working. The academic based team members were restricted from accessing University sites and thus their usual laboratories for 12 months and even the NHS based members were impacted by distancing policies and rotas, which limited use of certain machining equipment and usual face-to-face collaborative working. Project progress was aided by STH team members having unusually well-equipped home fabrication facilities and possessing the expertise to employ them effectively. Visits to collaborating sub-contractors in Chesterfield, Leeds and Durham to review requirements, materials and manufacturing options were not possible. Even simple logistical tasks such as moving materials and prototypes between partners became more onerous.

**Figure 5.**
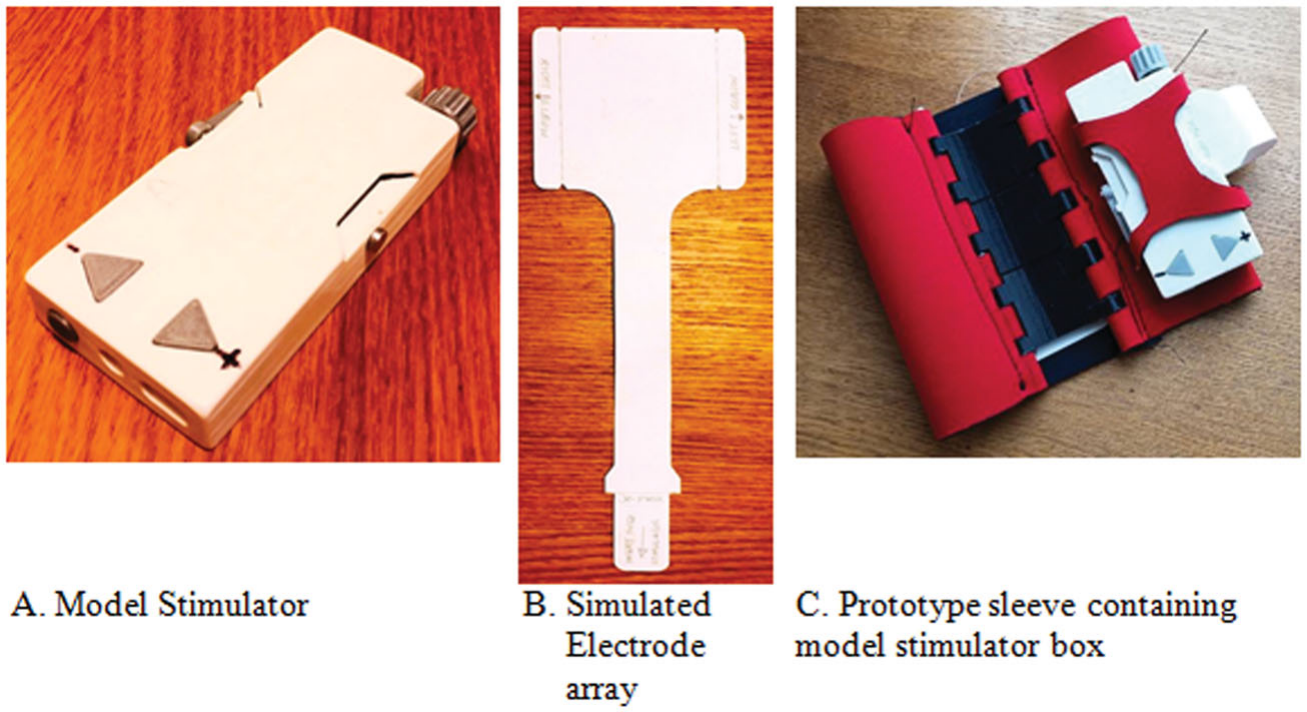
(A–C) Illustrative components parts.

Development work during the pandemic has focussed on the stimulator, electrode array, fit to arm and integration with arm sleeve components (illustrated in Figure 5). We have sought to enhance usability to enable use at home, and ideally one-handed operation by the stroke survivor. Figure 5 shows prototype models of the key component parts of the device; the stimulator box (A), a model of the electrode array that is inserted into the stimulator and will make direct contact with the arm (B), and the sleeve that holds the device in position on the arm (C). Other array models (not shown) were fabricated subsequently to closer represent the array’s behaviour and design.

In order to successfully administer the treatment it is necessary for the user to be able to:

1. Connect the electrode array to the stimulator box.
2. Put on and take off the arm sleeve.
3. Place and secure the electrode array over the correct location on the arm.
4. Switch the stimulator box on and off, and adjust the level of stimulation. (There will also be an auto-off, after treatment).

**Figure 6.**
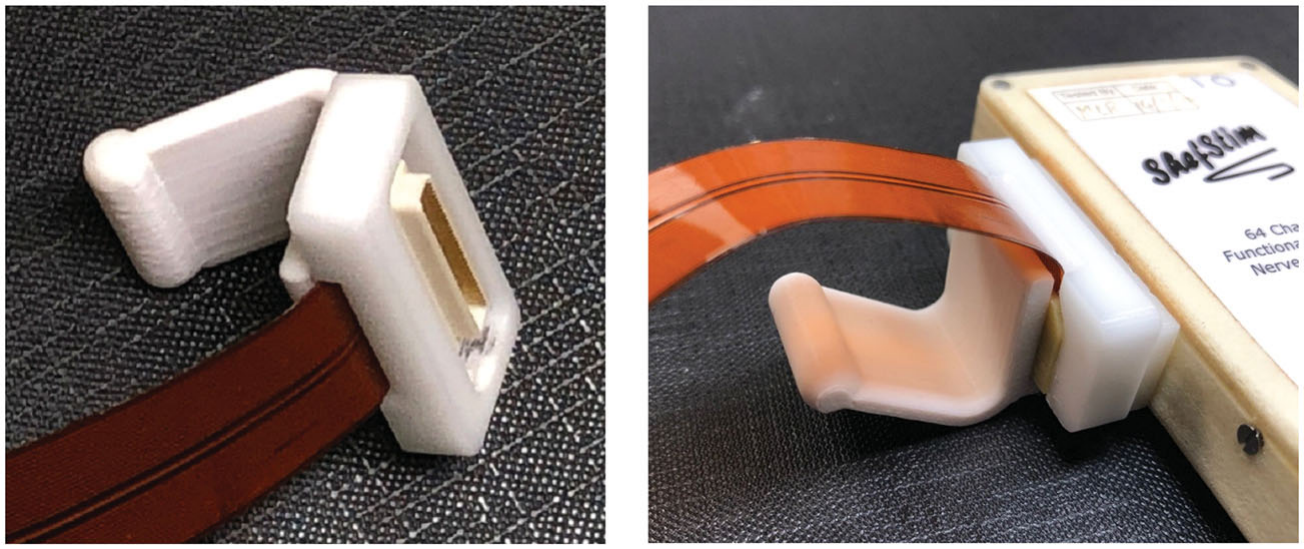
Push fit connector with additional grip as used in previous studies.

The previous studies utilised a miniature high density push-fit electrical connector for the electrode array which can be challenging to use effectively for someone with impaired dexterity and strength. These were found to be difficult for participants to attach and remove, even with the assistance of an additional grip attached (Figure 6). Although the frequency of needing to change arrays is expected to be limited, effort was made to improve the design for two-fold benefits:

1. Aid usability for the stroke survivors and carers.
2. Aid ease of manufacture by reducing the complexity of the soldering required (previously 0.5mm pitch 70 way connector to polyimide flexible circuit).

A significant degree of design effort was expended in moving the mechanical and electrical connectivity of the “connector” from the electrode array and integrating it within the body of the simulator. From a user perspective, this results in a requirement to simply introduce the “tail” of the electrode array into a slot with minimal insertion or closure force. A cam and lever mechanism was designed to lock the array securely – again with the aim of minimal force or dexterity required to insert the connector and operate the lever. The redesign of the connector had to balance the conflicting requirements of ease of use, functionality and safety. To avoid the risk of inadvertent connection of the patient-connected electrodes to a conductive surface, the electrode tail length is kept short and intended to be connected and assembled within the sleeve prior to donning.

IEC 60601-2-10 requires functional controls to be present on the stimulator box. The form of the controls for switching the device on and off, and adjusting the level of stimulation up and down for comfort was considered with two design options developed and presented to users through physical prototypes (Figure 5(A)).

Following prior user feedback regarding slow charging times, which could result in the inconvenient discovery of a partially discharged battery before intended usage, a design decision was made to improve several aspects of battery management. As a result speed of battery charging has been improved alongside an extended battery-life to reduce the required charging frequency from daily to weekly, introduction of a battery “fuel gauge,” and in addition, a mechanism has been added to remind users to charge the device.

### Adapted usability testing

The usability engineering methods applied had to be adapted to reflect the increased risk to stroke survivors presented by face-to-face group sessions, adherence to changing travel restrictions, access to facilities and social distancing measures. Although stroke was not a condition that necessarily required shielding, survivors often have other conditions placing them at greater risk of complications from COVID-19 infection [42]. Ideally preference and iterative usability testing of mock-ups and prototypes would have been undertaken face-to-face throughout the project allowing handling of artefacts and collaborative rapport to build between co-designers (including stroke survivors, carers, clinical specialists, engineering design team).

### Online working and data collection

As the research team were advised by their respective organisations to work from home where possible, and were not permitted to bring visitors into the work environment, this resulted in collaborative working and data collection making use of a range of online video-conferencing tools. Some platforms (e.g., Zoom) preferred by participants are not ones routinely permitted in the NHS. The research team included a stroke survivor who had lived experience of the condition, which proved very helpful through video usability reviews during the second lock-down to assess initial design options, prior to arranging wider feedback from users.

### Involving and engaging participants

Running preference and usability sessions online affected involvement of participants and reduced the number and the diversity of participants. The participant sample was restricted to those that could easily be reached, and who were well versed with remote online meeting.

Three healthcare professionals (who deliver FES clinic for patients with conditions including stroke and multiple sclerosis), were involved in usability assessments, as well as seven participants (5 stroke survivors and 2 spouses/informal carers) recruited from the Sheffield Stroke & Aphasia Group. The group meet monthly and were already meeting online due to the pandemic. The participants were further along (>6 months) in their post-stroke recovery than those most likely to benefit from SHAPES-based therapy, however, the pandemic related restrictions prevented access to patients soon after a stroke.

The approach to recruitment led to a potential bias towards stroke survivors with left-sided brain injury (right-arm weakness). Although there is evidence to suggest that such left-sided over-representative is reflective of left-sided symptoms being more easily recognised clinically at stroke presentation [43]. Left-sided injury is more associated with aphasia, a complex language and communication disorder resulting from damage to the language centres of the brain (experienced by 21–38% of acute stroke patients [44]). It can cause difficulties with speaking, understanding speech, and reading. A group of participants without aphasia could have led to underestimation of the challenges in communication and usability faced by users of the device, but ideally we would have been able to draw a more balanced participant group together to consider the usability.

### Provision of artefacts for user testing

In exploring usability, typically mock-ups and prototypes would be made to act as a discussion focus during product walkthroughs and user testing [18]. Non-functional and functional artefacts provide physical items that give users a sense of form and function, facilitating early changes during the development process. Previously we would have produced one-off items, which would have been reviewed collectively within a co-creation workshop, or used individually (and repeatedly) across multiple product walkthroughs. To support review in an online environment, the participants were each sent physical artefacts in advance of the sessions. The technical team undertook additional fabrication with multiple additive manufactured parts, sewn arm-sleeves and array mock-ups produced for each participant. Additive manufacturing utilising - now ubiquitously available - fused filament fabrication 3D printers made it possible to produce prototypes of the stimulators quickly and at low cost, thus enabling them to be treated as single-use disposable items. The design artefacts allowed users to assemble, handle and position the components as would be required for use.

Increased consideration was given to how the artefacts should be provided and explained to the participants. They were sent items in the post *via* the Sheffield Stroke Group ahead of the testing session; to maintain confidentiality address details were not shared with the project team. All artefacts were treated as disposable single-participant use only items and hard surfaces wiped with a chlorine dioxide based disinfectant (Tristel Jet) after handling by team members as per COVID-19 protocols.

### Design and delivery of the online sessions

The online sessions explored participant preferences and the usability of specific elements of the SHAPES design. The participants were able to handle the components in advance if they wished, and were then talked through the usage-case tasks during the session. To emulate traditional face to face sessions the online version had regular breaks, the sessions were broken down into meetings over consecutive days to prevent fatigue and session lengths were in line with the regular group meeting arrangements.

The D4D PPI team supported the development of an aphasia friendly format and pace, and the sessions were facilitated by a PPI specialist with prior experience of communication and working with stroke survivors. The language of the instructions, slide sets and usability questions and prompts were reviewed to ensure they suited participant comprehension and capabilities. Regular pauses to check understanding were used throughout each meeting. Verbal and written summaries of the discussions were given at the end of each meeting for shared understanding, and to allow opportunity for any clarification.

### Reviewing usability

**Figure 7.**
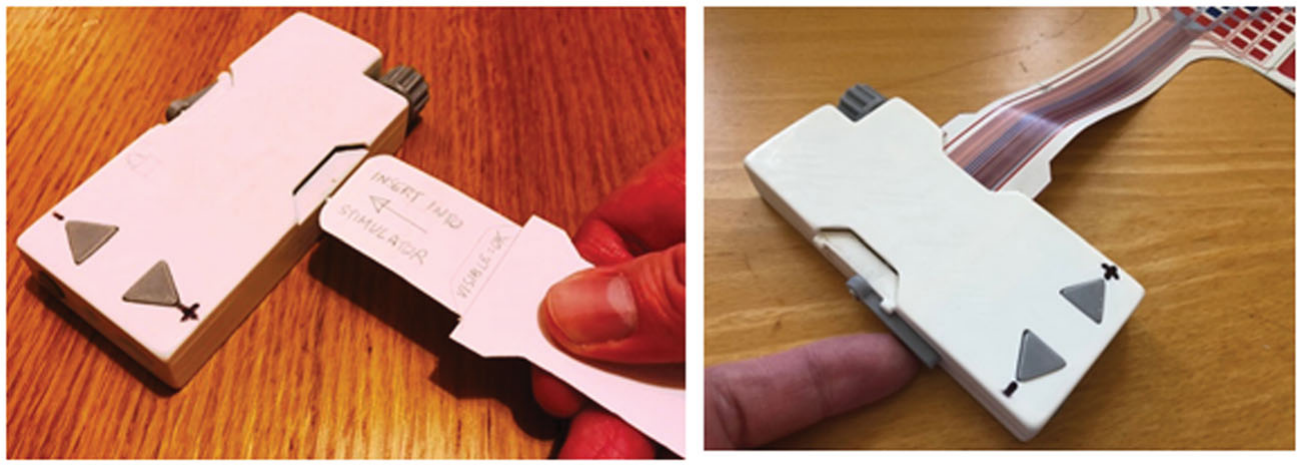
Electrode array mock-ups slotted into and secured in the model stimulator.

The usability sessions included consideration of the connection of the electrode array to the stimulator. This is achieved by inserting the smaller end of the array into the slot on the side of the stimulator (Figure 7). It can be connected either side, for left or right arm use. The participants were able to interact with their model to test the insertion of the array, and the lever which secures it in place. They gave individual feedback on the ease of use of the mechanism and discussed alternative design ideas.

**Figure 8.**
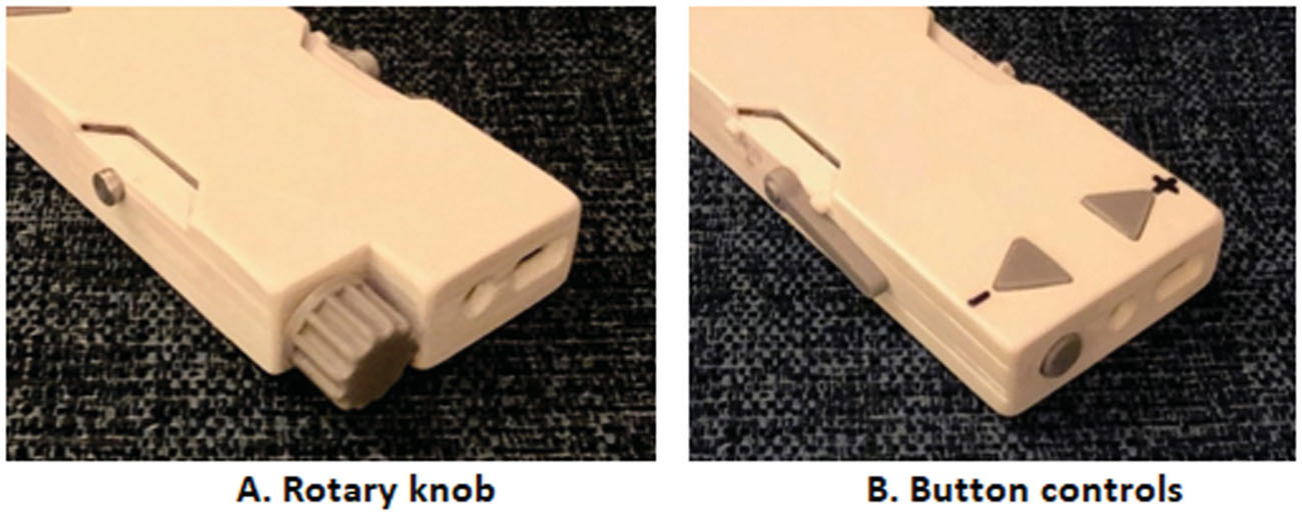
Options for stimulator control.

The mock-up stimulator box artefacts (Figure 8) enabled the participants to review and test two different control options for switching the stimulator on and off, and to turn the level of stimulation up and down, when worn on the arm (initially held in place with elasticated strapping).

One end of the box allowed testing of the up/down buttons; and the other testing of the rotary knob (with integrated push-button on/off function). Initial semi-structured interviews by the design team of three healthcare professionals helped narrow down to these two particular control options based on typical deficits experienced by patients and alleviate the need to send further options to the participants. During the session, the participants were able to indicate which they felt was easiest for them to understand and use, then suggest further design cues to guide correct usage. Despite an expectation that for clinical reasons, “up/down” buttons should be easier to manipulate, the wider context of usage resulted in a stronger preference for the rotary knob from the healthcare professionals and stroke survivors participating in the separate usability sessions. There are several possible reasons:

- Only needing to locate and manipulate one control could be perceived as easier than relocating between different and separate control buttons
- There could have been a degree of prior familiarity with a rotary knob for those who may previously have had experience of an FES device
- The buttons were aligned horizontally to accommodate the electronics contained within the casework. For a control that is essentially acting a “volume” control to adjust stimulation intensity up or down for comfort, a vertical arrangement of the buttons may have been perceived as more intuitive and presented a perhaps different view.

### Key design outputs that resulted from the feedback from user groups

1. Improved battery management of the stimulator
2. Decision to incorporate rotary control knob rather than buttons on the stimulator
3. Decision to include 2 supplementary graphics on the primary outcome measure – selected from 13 variants including proposals from users.
4. Redesign of stimulator connector and confirmation that the new design is usable one-handed.
5. Redesign of arm-sleeve and confirmation that the new design supports one-handed use and is acceptable for use within the trial

### Benefits and limitations

Online testing reduced the need for participant travel, potentially enabling participation by those with more significant disabilities, or without carer support. However, it did not result in access to ethnic or linguistic minority groups. Joining a virtual session, with both facilitators and participants in their own homes may have provided a greater sense of equality and participation from a friendly and familiar environment rather than a “professional” environment. The online session reduced the opportunity for informal interaction between people, and the ease of physical demonstration and observation of user: product interaction. However, we were able to easily record the session for later review by the design and engineering teams. The team was advised to restrict the number of researcher/facilitators present to a minimum so as not to place undue pressure on the participants. With the agreement of the participants the sessions were recorded for later review by team members who were not present. For future online sessions, more varied on-line approaches will be investigated. Examples include:

- Including meeting observers (muted and with cameras switched off) whose role is to assist in gauging reactions and to alert the facilitator/s discretely through use of private chat messages
- Making use of break-out rooms to offer parallel small groups for wider and longer discussions or for specific topic discussions– and be more akin to what would be done in a physical meeting.
- Offering more facilitators, and facilitators with different styles, knowledge of the devices and languages to suit differences in participant interests, understanding, language and diversity.
- Facilitating the engineering design team to have real-time observation of unscripted patient interaction with prototype devices, which is important in identifying circumstances that could lead to unintended misuse, and which thus present safety risks.

The online assessment of usability requires a change of approach in communication as the subtleties of non-verbal communication can be lost or mis-interpreted in a distanced environment. While a valuable resource, the online approach requires a high level of preparation and planning. Other potential challenges include:

- More time required to ensure understanding of the usability questions
- Potential for restricted “serial” discussions
- Potential to be harder to moderate depending on the participants present
- Potential to be harder to communicate empathy alongside “fact finding”
- Ensuring that people are listening to other participants’ views
- Balancing need for privacy and confidentiality with design team involvement

### Staff resources, collaboration and team work

The SHAPES project is led by an NHS Trust and focuses on developing a treatment for NHS patients. The research has been able to continue through COVID-19, but the context has increased the complexity of project management and delivery. The “working from home” requirement put in place through the pandemic phase affected iterative technical development and feedback cycles. The team had reduced access to laboratories, workshops, and printing facilities on NHS and University partner sites.

A number of the team members, stakeholders and participants have clinical roles within the healthcare system and continue to face the daily pressures of the pandemic. There has been necessary prioritisation of other work activities to meet competing organisational or patient needs with NHS staff members redeployed to COVID related duties. Colleagues working for manufacturing companies and universities have also had to adjust their working practices and adapt to changing workloads, and distribution of responsibilities. Team members have had COVID-19, had to self-isolate due to contact tracing, been subject to national and local lock-downs, as well as balancing home working with home schooling.

To date (October 2021), the full team has not being able to meet together face-to face due to travel restrictions and social distancing measures, meeting virtually as a large group, and with smaller face to face meetings when, and where possible. The lack of predictability of NHS demands, staff availability, and access to patients, facilities and materials has required agile project management and a robust risk management to adapt to evolving circumstances and demands and ensure compliance to national, NHS and local Trust response plans and guidance.

## Discussion and conclusions

Despite the many challenges the pandemic has provoked, it has also necessitated undertaking usability engineering differently; to employ a wider range of methods, to develop solutions, and potentially to offer vulnerable lay users more flexible approaches to provide their valuable insights.

During the pandemic other researchers have taken various approaches to minimise user risk during usability engineering. This has led to increased data collection and testing online *via* video conferencing platforms. Most recent reports of online usability testing relate to the use of physical interventions (e.g., walking rehabilitation) and computer-based/digital systems (e.g., games, apps) rather than use of physical products [45–47]. Others have explored the use of virtual and augmented reality solutions to establish a surrogate relationship between participants and a facilitating researcher through video conferencing [48].

Unlike with the SHAPES project, we have not yet identified another study where prototypes have been distributed during the pandemic for remote user testing.

The ShefStim APS is a novel electronic rehabilitation device, expected to provide benefits by offering a simple self or carer-managed intervention that could be deployed in the community early after stroke to treat post-stroke elbow spasticity. It also has the potential to be used for other joints and other neurological conditions like multiple sclerosis, traumatic brain injury, spinal cord injury and cerebral palsy. If proven effective, the SHAPES stimulation technique would allow more therapy to be delivered in the community without the significant side-effects and extra cost. Given the potential benefits to patient care, rather than delay the project start, or significantly alter the project plan due to the global pandemic, a range of strategies were implemented to allow timely delivery without compromising design and usability. A summary of the some of the challenges experienced and the solutions used are shown below in Table 2.

**Table 2.** A summary of challenges faced and strategies employed in response.

Challenge	Opportunity/solution
Prototype development	
Working from home arrangements affected technical development with reduced access to labs, workshops, fabrication facilities on NHS and University sites Supply and logistical delays: electronic components became unavailable or with lead times of up to a year and other consumables more time-consuming to source. Procurement issues necessitated several unplanned engineering re-designs to accommodate the shortages. Design of non-functional prototypes is informed by the parallel design development of the actual device. Delays in either design or usability sessions impact upon each other.	Made and utilised “paperware” early on to demonstrate key concepts & created standardised video walk-throughs Team members utilised personal fabrication and high-performance computing facilities at home e.g., 3D printers, CAD modelling and simulation, industrial sewing machines and existing material swatches to create prototype functional models and arm-sleeves Team members with access to facilities undertook extra design work that would not usually have been their responsibility.
Preference and iterative usability testing of prototypes with end-users	
Face to face user research was not possible due to travel restrictions, social distancing and the vulnerability of stroke survivors and of some team members Online data collection potentially reduced diversity of patients and may have limited inclusion for those without internet access or digital capabilities. Meeting participant needs while meeting and working online Infection control measures to be addressed to enable patients to handle prototypes Online meetings lack the social aspect that many stroke survivors enjoy	Online video platform such as MS Teams and Zoom were employed which enabled participants with internet access to join – who might otherwise might not have wished to attend a physical meeting due to fatigue or the stress of travel With participant consent, offered the opportunity to record and review usability sessions.
Staff resources, collaboration and team work	
Team members ill and / or isolating Availability of, and pressure on NHS colleagues as collaborators and participants Necessary prioritisation of other work activities to meet competing organisational or patient needs Team members home working and home schooling Absence of face to face team meetings and team working due to travel restrictions and social distancing measures Uncertainty and changing national and local guidelines	Imbued a collective willingness to overcome adversity (often referred to in the UK as “*Dunkirk spirit*”). Team members went “the extra mile” to overcome difficulties. Made the most of the online facilities that were available Created bespoke demonstration videos, developed illustrated information sheets and aphasia-friendly versions of information provided.

For a device intended to be used by stroke survivors and lay carers in a home environment, the challenges of effective PPI and usability development are magnified, and further exacerbated in the context of a global pandemic. The impact of COVID-19 necessitated online rather than physical testing sessions. We have adapted to online working and successfully reviewed usability of prototypes through distanced means. It has not been ideal. These new approaches led to additional technical and logistical effort in preparing and posting out one-off design prototypes suitable for sharing with participants. Changes in approach slowed progress compared with more typical face-to-face meetings and workshops. However, the effort worked well for our participants who did not need to travel, or feel anxious about social contact and it reduced the need to arrange meeting venues etc. We do still hope to be able to return to physical testing and co-design in the future; the virtual approach does limit “softer” interactions including the conveying of empathetic discussions and useful conversations as well as limiting the number of people who can effectively be included for design reviews.

It is important to note that researchers should be flexible in their usability approach according to the project requirements and the needs of the patient population likely to take part in that group. While there is generic online guidance relating to user involvement available from organisations such as the NIHR’s Centre for Engagement and Dissemination Centre [49], the European Patients’ Academy on Therapeutic Innovation (EUPATI) [50] and The King’s Fund [51], such guidance is primarily focussed on the patient public involvement aspects of including users in healthcare research. In addition, researchers are recommended to utilise the guidance and standards discussed in Table 1 for the specifics of medical device development and in their considerations when planning usability evaluations.

The stimulator hardware is being finalised prior to the start of a randomised clinical trial due to commence in the Spring of 2022. Future co-design activity will focus on the re-development of an arm–sleeve. Our focus will be on ensuring that the sleeve delivers two functions effectively. It should maintain a constant, even contact of the stimulating end of the electrode array (overlaid with hydrogel) with the user’s arm, and, hold the stimulator securely on the outside of the sleeve, while protecting it from impact. The sleeve needs to secure the array on the upper arm - tightly enough for continuous direct contact to the skin without leading to discomfort or skin damage. The intention is that the sleeve can be fitted and secured independently and using only one hand. To achieve this, further usability engineering plus future co-design will be critical.
